# [μ-*N*,*N*,*N*′,*N*′-Tetra­kis(2-pyridyl­meth­yl)butane-1,4-diamine]­bis­[dibromidocopper(II)]

**DOI:** 10.1107/S1600536810034537

**Published:** 2010-09-04

**Authors:** Mark Bartholomä, Hoi Cheung, Jon Zubieta

**Affiliations:** a Department of Chemistry, Syracuse University, Syracuse, New York 13244, USA

## Abstract

The title dinuclear copper complex, [Cu_2_Br_4_(C_28_H_32_N_6_)], is located on an inversion center. The unique Cu^II^ ion is in a slightly distorted square-pyramidal environment in which the N atoms of a dipicolyl­amine group and a bromide ligand form the basal plane. The apical site is occupied by a second Br atom. While the Cu—N distances involving the pyridine N atoms are the same within experimental error, the Cu—N distance involving the tertiary N atom is slightly elongated. Due to the typical Jahn–Teller distortion of copper(II) complexes, the apical Cu—Br distance is elongated.

## Related literature

For crystallographic data of tetra­kis­(pyridin-2-yl-meth­yl)alkyl-diamine compounds, see: Fujihara *et al.* (2004 ▶); Mam­banda *et al.* (2007 ▶). For the superoxide dismutase activity of iron complexes, see: Tamura *et al.* (2000 ▶). For dinuclear Pt complexes of similar ligands, see: Ertürk *et al.* (2007 ▶). For the use of the dipicolyl­amine moiety for binding of the *M*(CO)_3_ core (*M* = Re,^99*m*^Tc), see: Bartholomä *et al.* (2009 ▶). For crystal structures closely related to the title compound, see: Bartholomä *et al.* (2010*a* ▶,*b* ▶,*c* ▶,*d* ▶).
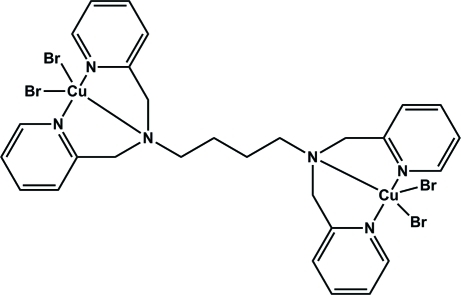


## Experimental

### 

#### Crystal data

[Cu_2_Br_4_(C_28_H_32_N_6_)]
*M* *_r_* = 899.32
Monoclinic, 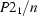

*a* = 8.8613 (6) Å
*b* = 14.249 (1) Å
*c* = 11.9488 (9) Å
β = 98.588 (2)°
*V* = 1491.80 (18) Å^3^
*Z* = 2
Mo *K*α radiation
μ = 6.81 mm^−1^
*T* = 90 K
0.18 × 0.12 × 0.08 mm

#### Data collection

Bruker APEX CCD diffractometer
Absorption correction: multi-scan (*SADABS*; Bruker, 1998 ▶) *T*_min_ = 0.374, *T*_max_ = 0.612
14513 measured reflections
3623 independent reflections
3171 reflections with *I* > 2σ(*I*)
*R*_int_ = 0.052

#### Refinement

*R*[*F* ^2^ > 2σ(*F* ^2^)] = 0.074
*wR*(*F* ^2^) = 0.147
*S* = 1.38
3623 reflections
181 parameters
H-atom parameters constrained
Δρ_max_ = 1.38 e Å^−3^
Δρ_min_ = −0.85 e Å^−3^

### 

Data collection: *SMART* (Bruker, 1998 ▶); cell refinement: *SAINT* (Bruker, 1998 ▶); data reduction: *SAINT*; program(s) used to solve structure: *SHELXS97* (Sheldrick, 2008 ▶); program(s) used to refine structure: *SHELXL97* (Sheldrick, 2008 ▶); molecular graphics: *DIAMOND* (Brandenburg & Putz, 1999 ▶); software used to prepare material for publication: *SHELXTL* (Sheldrick, 2008 ▶).

## Supplementary Material

Crystal structure: contains datablocks I, global. DOI: 10.1107/S1600536810034537/lh5106sup1.cif

Structure factors: contains datablocks I. DOI: 10.1107/S1600536810034537/lh5106Isup2.hkl

Additional supplementary materials:  crystallographic information; 3D view; checkCIF report

## Figures and Tables

**Table 1:** Selected bond lengths (Å)

Cu1—N2	2.015 (6)
Cu1—N3	2.019 (6)
Cu1—N1	2.053 (5)
Cu1—Br2	2.4099 (11)
Cu1—Br1	2.7045 (11)

**Fig. 1.**
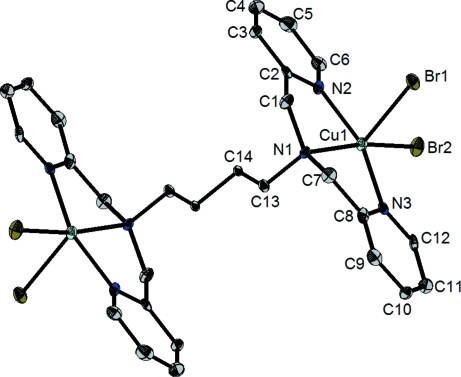
The crystal structure of the title complex. The displacement ellipsoids are drawn at 50% probability level. Hydrogen atoms are omitted for clarity. Unlabeled atoms are related by the symmetry code (-x + 1, -y + 1, -z + 1).

## supplementary crystallographic information

### Comment

The described ligand *N^1^*,*N^1^*,*N^4^*,*N^4^*-tetrakis(pyridin-2-ylmethyl)butane-1,4-diamine has been used as starting material for hydrothermal synthesis of metal-organic transition metal/molybdateoxide frameworks in the principal author's laboratory (Bartholomä, unpublished results). The dipicolylamine moiety has originally been used in our laboratory as metal chelating entity for binding of the *M*(CO)_3_ core (*M* = Re,^99^*^m^*Tc) for radiopharmaceutical purposes. A different coordination mode has been observed for the *M*(CO)_3_ core in which the dipicolylamine metal chelate is coordinated in a facial manner (Bartholomä, 2009).

The title complex was prepared as part of a series with different cadmium and copper salts to study the coordination properties of the ligand with these metals without the interaction of metaloxide clusters (Bartholomä, 2010*a*,b,c). The crystalline sample obtained with copper chloride as metal source and *N^1^*,*N^1^*,*N^5^*,*N^5^*-tetrakis(pyridin-2-ylmethyl)pentane-1,5-diamine gave a structurally similar compound with a distorted square pyramidal coordination geometry of the copper atoms as observed for the described complex. The Cu—N_py_ distances of 1.986 (4) Å and 1.996 (4), and a Cu—N*_tert_* distance of 2.076 (4) Å (Bartholomä, 2010d).

Crystal structures of the ligands *N^1^*,*N^1^*,*N^3^*,*N^3^*-tetrakis(2-pyridiniomethyl)-1,3-diaminopropane and *N^1^*,*N^1^*,*N^4^*,*N^4^*-tetrakis(pyridin-2-ylmethyl)butane-1,4-diamine have been described recently (Fujihara, 2004; Mambanda, 2007). Superoxide dismutase activity of iron(II) complexes of *N^1^*,*N^1^*,*N^3^*,*N^3^*-tetrakis(2-pyridiniomethyl)-1,3-diaminopropane and related ligands has been investigated by Tamura *et al.* (2000). Studies on the thermodynamic and kinetic behaviour of the reaction of platinum(II) complexes of higher ligand homologues with chloride have been performed by Ertürk *et al.* (2007).

### Experimental

*N^1^*,*N^1^*,*N^4^*,*N^4^*-tetrakis(pyridin-2-ylmethyl)butane-1,4-diamine. An amount of 1.00 g (11.34 mmol) 1,4-diaminobutane was dissolved in 30 ml anhydrous dichloroethane under an inert atmosphere (argon) followed by the addition of 4.55 ml (47.65 mmol) pyridine-2-carboxaldehyde. The mixture was stirred for 15 min at r.t. and then cooled with an ice bath prior to the portionwise addition of 14.43 g (68.06 mmol) sodium triacetoxyborohydride (gas evolution, exothermic reaction). The reaction was stirred overnight allowing the temperature slowly to rise to room temperature. The reaction was quenched by the dropwise addition of saturated sodium bicarbonate solution and stirring was continued until the gas evolution ceased. The mixture was separated and the organic layer was further washed with saturated sodium bicarbonate solution, water and brine. The organic phase was dried with anhydrous sodium sulfate, filtered and the solvent removed under reduced pressure. The crude reaction mixture was then purified by silica gel column chromatography starting with chloroform and increasing gradient to chloroform:methanol 10:1 (*v*/*v*). Yield: 4.02 g (78%). ^1^H NMR (CDCl_3_): *δ* = 8.40 (m, 4H), 7.51 (m, 4H), 7.39 (d, J = 7.81 Hz, 4H), 7.02 (m, 4H), 3.67 (s, 8H), 2.39 (m, 4H), 1.42 (m, 4H) p.p.m..

Synthesis of metal complex. To 2 ml of an aqueous solution of copper bromide, two equivalents (50 mg, 0.11 mmol) of *N^1^*,*N^1^*,*N^4^*,*N^4^*-tetrakis(pyridin-2-ylmethyl)butane-1,4-diamine in 2 ml methanol were added followed by the addition of 2 ml *N*,*N*-dimethylformamide. Single crystals were obtained after a week by slow evaporation of the solvents at room temperature.

### Refinement

All the H atoms were placed in idealized positions and refined in a riding-model approximation with *C*—H_aryl_ = 0.95 Å, *C*—H_methyl_ = 0.98Å and *C*—H_methylene_ = 0.99Å and *U*_iso_(H) = 1.5*U*_eq_(C_methyl_) and 1.2*U*_eq_(C_methylene/aryl_).

### Crystal data

**Table:**

[Cu_2_Br_4_(C_28_H_32_N_6_)]	*F*(000) = 880
*M**_r_* = 899.32	*D*_x_ = 2.002 Mg m^−^^3^
Monoclinic, *P*2_1_/*n*	Mo *K*α radiation, λ = 0.71073 Å
Hall symbol: -P 2yn	Cell parameters from 2666 reflections
*a* = 8.8613 (6) Å	θ = 2.7–27.2°
*b* = 14.249 (1) Å	µ = 6.81 mm^−^^1^
*c* = 11.9488 (9) Å	*T* = 90 K
β = 98.588 (2)°	Plates, green
*V* = 1491.80 (18) Å^3^	0.18 × 0.12 × 0.08 mm
*Z* = 2	

### Data collection

**Table:**

Bruker APEX CCD diffractometer	3623 independent reflections
Radiation source: fine-focus sealed tube	3171 reflections with *I* > 2σ(*I*)
graphite	*R*_int_ = 0.052
Detector resolution: 512 pixels mm^-1^	θ_max_ = 28.1°, θ_min_ = 2.2°
φ and ω scans	*h* = −10→11
Absorption correction: multi-scan (*SADABS*; Bruker, 1998)	*k* = −18→18
*T*_min_ = 0.374, *T*_max_ = 0.612	*l* = −15→15
14513 measured reflections	

### Refinement

**Table:**

Refinement on *F*^2^	Primary atom site location: structure-invariant direct methods
Least-squares matrix: full	Secondary atom site location: difference Fourier map
*R*[*F*^2^ > 2σ(*F*^2^)] = 0.074	Hydrogen site location: inferred from neighbouring sites
*wR*(*F*^2^) = 0.147	H-atom parameters constrained
*S* = 1.38	*w* = 1/[σ^2^(*F*_o_^2^) + (0.042*P*)^2^ + 10.8322*P*] where *P* = (*F*_o_^2^ + 2*F*_c_^2^)/3
3623 reflections	(Δ/σ)_max_ = 0.001
181 parameters	Δρ_max_ = 1.38 e Å^−^^3^
0 restraints	Δρ_min_ = −0.85 e Å^−^^3^

### Special details

**Table:**

Geometry. All e.s.d.'s (except the e.s.d. in the dihedral angle between two l.s. planes) are estimated using the full covariance matrix. The cell e.s.d.'s are taken into account individually in the estimation of e.s.d.'s in distances, angles and torsion angles; correlations between e.s.d.'s in cell parameters are only used when they are defined by crystal symmetry. An approximate (isotropic) treatment of cell e.s.d.'s is used for estimating e.s.d.'s involving l.s. planes.
Refinement. Refinement of *F*^2^ against ALL reflections. The weighted *R*-factor *wR* and goodness of fit *S* are based on *F*^2^, conventional *R*-factors *R* are based on *F*, with *F* set to zero for negative *F*^2^. The threshold expression of *F*^2^ > σ(*F*^2^) is used only for calculating *R*-factors(gt) *etc*. and is not relevant to the choice of reflections for refinement. *R*-factors based on *F*^2^ are statistically about twice as large as those based on *F*, and *R*- factors based on ALL data will be even larger.

### Fractional atomic coordinates and isotropic or equivalent isotropic displacement parameters (Å^2^)

**Table:**

	*x*	*y*	*z*	*U*_iso_*/*U*_eq_	
Cu1	0.35669 (10)	0.75522 (6)	0.35899 (7)	0.0111 (2)	
Br1	0.21437 (9)	0.82045 (5)	0.15805 (6)	0.01711 (19)	
Br2	0.29274 (9)	0.87831 (5)	0.48204 (6)	0.01847 (19)	
N1	0.4360 (6)	0.6374 (4)	0.2878 (5)	0.0087 (11)	
N2	0.1789 (7)	0.6693 (4)	0.3702 (5)	0.0101 (11)	
N3	0.5695 (6)	0.8000 (4)	0.3456 (5)	0.0099 (11)	
C1	0.2983 (8)	0.5899 (5)	0.2279 (6)	0.0138 (14)	
H1A	0.3214	0.5230	0.2157	0.017*	
H1B	0.2676	0.6195	0.1530	0.017*	
C2	0.1707 (8)	0.5972 (5)	0.2964 (6)	0.0118 (13)	
C3	0.0522 (8)	0.5333 (5)	0.2869 (6)	0.0161 (15)	
H3	0.0470	0.4837	0.2334	0.019*	
C4	−0.0587 (9)	0.5423 (5)	0.3564 (7)	0.0208 (16)	
H4	−0.1415	0.4994	0.3507	0.025*	
C5	−0.0474 (9)	0.6153 (6)	0.4350 (7)	0.0214 (16)	
H5	−0.1201	0.6219	0.4854	0.026*	
C6	0.0718 (8)	0.6775 (5)	0.4377 (6)	0.0159 (14)	
H6	0.0784	0.7284	0.4896	0.019*	
C7	0.5359 (8)	0.6749 (5)	0.2094 (6)	0.0136 (14)	
H7A	0.4729	0.7002	0.1406	0.016*	
H7B	0.6010	0.6242	0.1863	0.016*	
C8	0.6340 (8)	0.7514 (5)	0.2682 (6)	0.0132 (14)	
C9	0.7788 (9)	0.7733 (5)	0.2435 (6)	0.0169 (15)	
H9	0.8206	0.7398	0.1866	0.020*	
C10	0.8606 (8)	0.8445 (5)	0.3033 (6)	0.0167 (15)	
H10	0.9603	0.8600	0.2890	0.020*	
C11	0.7950 (9)	0.8930 (5)	0.3845 (6)	0.0186 (15)	
H11	0.8492	0.9421	0.4267	0.022*	
C12	0.6489 (9)	0.8689 (5)	0.4032 (6)	0.0147 (14)	
H12	0.6041	0.9024	0.4587	0.018*	
C13	0.5282 (8)	0.5732 (5)	0.3704 (6)	0.0111 (13)	
H13A	0.6209	0.6073	0.4052	0.013*	
H13B	0.5620	0.5193	0.3281	0.013*	
C14	0.4471 (7)	0.5353 (4)	0.4650 (5)	0.0096 (13)	
H14A	0.3506	0.5042	0.4323	0.011*	
H14B	0.4224	0.5875	0.5139	0.011*	

### Atomic displacement parameters (Å^2^)

**Table:**

	*U*^11^	*U*^22^	*U*^33^	*U*^12^	*U*^13^	*U*^23^
Cu1	0.0124 (4)	0.0090 (4)	0.0126 (4)	0.0003 (3)	0.0041 (3)	−0.0005 (3)
Br1	0.0196 (4)	0.0162 (4)	0.0155 (3)	0.0032 (3)	0.0024 (3)	0.0062 (3)
Br2	0.0249 (4)	0.0131 (3)	0.0198 (4)	−0.0001 (3)	0.0110 (3)	−0.0037 (3)
N1	0.009 (3)	0.008 (3)	0.011 (3)	−0.002 (2)	0.004 (2)	0.000 (2)
N2	0.014 (3)	0.009 (3)	0.008 (2)	−0.002 (2)	0.003 (2)	0.003 (2)
N3	0.010 (3)	0.009 (3)	0.011 (3)	0.002 (2)	0.003 (2)	0.004 (2)
C1	0.018 (4)	0.012 (3)	0.012 (3)	−0.003 (3)	0.003 (3)	−0.006 (3)
C2	0.013 (3)	0.010 (3)	0.011 (3)	0.003 (3)	−0.003 (3)	0.005 (2)
C3	0.017 (4)	0.010 (3)	0.018 (3)	−0.002 (3)	−0.006 (3)	−0.002 (3)
C4	0.013 (4)	0.019 (4)	0.032 (4)	−0.002 (3)	0.005 (3)	0.005 (3)
C5	0.014 (4)	0.027 (4)	0.025 (4)	0.001 (3)	0.007 (3)	0.009 (3)
C6	0.014 (4)	0.018 (4)	0.015 (3)	0.003 (3)	0.003 (3)	0.001 (3)
C7	0.014 (4)	0.016 (3)	0.013 (3)	0.001 (3)	0.009 (3)	−0.004 (3)
C8	0.019 (4)	0.011 (3)	0.010 (3)	−0.001 (3)	0.003 (3)	0.005 (3)
C9	0.018 (4)	0.019 (4)	0.016 (3)	0.003 (3)	0.007 (3)	0.005 (3)
C10	0.011 (3)	0.015 (3)	0.024 (4)	−0.004 (3)	0.002 (3)	0.009 (3)
C11	0.019 (4)	0.015 (3)	0.020 (4)	−0.001 (3)	−0.002 (3)	0.001 (3)
C12	0.021 (4)	0.008 (3)	0.016 (3)	0.002 (3)	0.003 (3)	0.007 (3)
C13	0.010 (3)	0.010 (3)	0.015 (3)	0.002 (3)	0.005 (3)	0.004 (2)
C14	0.007 (3)	0.008 (3)	0.013 (3)	0.000 (2)	0.001 (2)	0.005 (2)

### Geometric parameters (Å, °)

**Table:**

Cu1—N2	2.015 (6)	C5—C6	1.375 (11)
Cu1—N3	2.019 (6)	C5—H5	0.9500
Cu1—N1	2.053 (5)	C6—H6	0.9500
Cu1—Br2	2.4099 (11)	C7—C8	1.502 (10)
Cu1—Br1	2.7045 (11)	C7—H7A	0.9900
N1—C7	1.482 (8)	C7—H7B	0.9900
N1—C1	1.482 (9)	C8—C9	1.394 (10)
N1—C13	1.495 (8)	C9—C10	1.382 (11)
N2—C6	1.340 (9)	C9—H9	0.9500
N2—C2	1.349 (9)	C10—C11	1.388 (11)
N3—C12	1.337 (9)	C10—H10	0.9500
N3—C8	1.349 (9)	C11—C12	1.389 (11)
C1—C2	1.496 (10)	C11—H11	0.9500
C1—H1A	0.9900	C12—H12	0.9500
C1—H1B	0.9900	C13—C14	1.526 (9)
C2—C3	1.381 (10)	C13—H13A	0.9900
C3—C4	1.384 (11)	C13—H13B	0.9900
C3—H3	0.9500	C14—C14^i^	1.536 (12)
C4—C5	1.395 (12)	C14—H14A	0.9900
C4—H4	0.9500	C14—H14B	0.9900
			
N2—Cu1—N3	161.0 (2)	C6—C5—H5	120.8
N2—Cu1—N1	81.4 (2)	C4—C5—H5	120.8
N3—Cu1—N1	81.0 (2)	N2—C6—C5	122.7 (7)
N2—Cu1—Br2	98.29 (16)	N2—C6—H6	118.7
N3—Cu1—Br2	97.22 (16)	C5—C6—H6	118.7
N1—Cu1—Br2	166.96 (16)	N1—C7—C8	109.0 (5)
N2—Cu1—Br1	90.12 (16)	N1—C7—H7A	109.9
N3—Cu1—Br1	97.95 (15)	C8—C7—H7A	109.9
N1—Cu1—Br1	93.22 (16)	N1—C7—H7B	109.9
Br2—Cu1—Br1	99.82 (4)	C8—C7—H7B	109.9
C7—N1—C1	112.7 (5)	H7A—C7—H7B	108.3
C7—N1—C13	108.6 (5)	N3—C8—C9	121.9 (7)
C1—N1—C13	111.7 (5)	N3—C8—C7	114.7 (6)
C7—N1—Cu1	103.9 (4)	C9—C8—C7	123.4 (6)
C1—N1—Cu1	105.4 (4)	C10—C9—C8	118.9 (7)
C13—N1—Cu1	114.4 (4)	C10—C9—H9	120.6
C6—N2—C2	119.0 (6)	C8—C9—H9	120.6
C6—N2—Cu1	128.2 (5)	C9—C10—C11	119.0 (7)
C2—N2—Cu1	112.7 (4)	C9—C10—H10	120.5
C12—N3—C8	119.0 (6)	C11—C10—H10	120.5
C12—N3—Cu1	128.1 (5)	C10—C11—C12	119.2 (7)
C8—N3—Cu1	112.9 (5)	C10—C11—H11	120.4
N1—C1—C2	109.8 (5)	C12—C11—H11	120.4
N1—C1—H1A	109.7	N3—C12—C11	122.0 (7)
C2—C1—H1A	109.7	N3—C12—H12	119.0
N1—C1—H1B	109.7	C11—C12—H12	119.0
C2—C1—H1B	109.7	N1—C13—C14	115.7 (5)
H1A—C1—H1B	108.2	N1—C13—H13A	108.4
N2—C2—C3	121.5 (7)	C14—C13—H13A	108.4
N2—C2—C1	116.0 (6)	N1—C13—H13B	108.4
C3—C2—C1	122.5 (6)	C14—C13—H13B	108.4
C2—C3—C4	119.2 (7)	H13A—C13—H13B	107.4
C2—C3—H3	120.4	C13—C14—C14^i^	108.6 (7)
C4—C3—H3	120.4	C13—C14—H14A	110.0
C3—C4—C5	119.2 (7)	C14^i^—C14—H14A	110.0
C3—C4—H4	120.4	C13—C14—H14B	110.0
C5—C4—H4	120.4	C14^i^—C14—H14B	110.0
C6—C5—C4	118.3 (7)	H14A—C14—H14B	108.3
			
N2—Cu1—N1—C7	151.6 (4)	Cu1—N2—C2—C3	−176.6 (5)
N3—Cu1—N1—C7	−35.6 (4)	C6—N2—C2—C1	−177.1 (6)
Br2—Cu1—N1—C7	−118.9 (7)	Cu1—N2—C2—C1	4.5 (7)
Br1—Cu1—N1—C7	62.0 (4)	N1—C1—C2—N2	24.0 (8)
N2—Cu1—N1—C1	32.9 (4)	N1—C1—C2—C3	−154.9 (6)
N3—Cu1—N1—C1	−154.3 (4)	N2—C2—C3—C4	−1.4 (10)
Br2—Cu1—N1—C1	122.4 (7)	C1—C2—C3—C4	177.4 (7)
Br1—Cu1—N1—C1	−56.7 (4)	C2—C3—C4—C5	−0.5 (11)
N2—Cu1—N1—C13	−90.2 (4)	C3—C4—C5—C6	2.1 (11)
N3—Cu1—N1—C13	82.6 (4)	C2—N2—C6—C5	−0.2 (10)
Br2—Cu1—N1—C13	−0.7 (10)	Cu1—N2—C6—C5	178.0 (5)
Br1—Cu1—N1—C13	−179.8 (4)	C4—C5—C6—N2	−1.7 (11)
N3—Cu1—N2—C6	137.8 (7)	C1—N1—C7—C8	158.0 (6)
N1—Cu1—N2—C6	160.1 (6)	C13—N1—C7—C8	−77.8 (7)
Br2—Cu1—N2—C6	−6.7 (6)	Cu1—N1—C7—C8	44.4 (6)
Br1—Cu1—N2—C6	−106.7 (6)	C12—N3—C8—C9	−2.4 (10)
N3—Cu1—N2—C2	−44.0 (9)	Cu1—N3—C8—C9	177.9 (5)
N1—Cu1—N2—C2	−21.7 (4)	C12—N3—C8—C7	179.3 (6)
Br2—Cu1—N2—C2	171.5 (4)	Cu1—N3—C8—C7	−0.3 (7)
Br1—Cu1—N2—C2	71.6 (4)	N1—C7—C8—N3	−30.7 (8)
N2—Cu1—N3—C12	−136.2 (7)	N1—C7—C8—C9	151.1 (6)
N1—Cu1—N3—C12	−158.5 (6)	N3—C8—C9—C10	2.5 (10)
Br2—Cu1—N3—C12	8.4 (6)	C7—C8—C9—C10	−179.4 (7)
Br1—Cu1—N3—C12	109.4 (5)	C8—C9—C10—C11	−1.1 (10)
N2—Cu1—N3—C8	43.4 (9)	C9—C10—C11—C12	−0.2 (10)
N1—Cu1—N3—C8	21.0 (4)	C8—N3—C12—C11	1.1 (10)
Br2—Cu1—N3—C8	−172.0 (4)	Cu1—N3—C12—C11	−179.4 (5)
Br1—Cu1—N3—C8	−71.0 (4)	C10—C11—C12—N3	0.2 (10)
C7—N1—C1—C2	−151.4 (6)	C7—N1—C13—C14	174.3 (6)
C13—N1—C1—C2	86.1 (7)	C1—N1—C13—C14	−60.8 (7)
Cu1—N1—C1—C2	−38.8 (6)	Cu1—N1—C13—C14	58.8 (7)
C6—N2—C2—C3	1.8 (10)	N1—C13—C14—C14^i^	175.1 (6)

Symmetry codes: (i) −*x*+1, −*y*+1, −*z*+1.
